# Infant Gut Microbial Metagenome Mining of α-l-Fucosidases with Activity on Fucosylated Human Milk Oligosaccharides and Glycoconjugates

**DOI:** 10.1128/spectrum.01775-22

**Published:** 2022-08-09

**Authors:** Eva M. Moya-Gonzálvez, Nazaret Peña-Gil, Antonio Rubio-del-Campo, José M. Coll-Marqués, Roberto Gozalbo-Rovira, Vicente Monedero, Jesús Rodríguez-Díaz, María J. Yebra

**Affiliations:** a Laboratorio de Bacterias Lácticas y Probióticos, Departamento de Biotecnología de Alimentos, Instituto de Agroquímica y Tecnología de Alimentos (IATA-CSIC), Valencia, Spain; b Departamento de Microbiología, Facultad de Medicina, Universidad de Valencia, Valencia, Spain; c INCLIVA, Instituto de Investigación Sanitaría del Hospital Clínico de Valencia, Valencia, Spain; University of Nevada, Reno

**Keywords:** α-l-fucosidase, metagenome, microbiota, infant gut, human milk oligosaccharides, histo-blood group antigens, glycoproteins

## Abstract

The gastrointestinal microbiota members produce α-l-fucosidases that play key roles in mucosal, human milk, and dietary oligosaccharide assimilation. Here, 36 open reading frames (ORFs) coding for putative α-l-fucosidases belonging to glycosyl hydrolase family 29 (GH29) were identified through metagenome analysis of breast-fed infant fecal microbiome. Twenty-two of those ORFs showed a complete coding sequence with deduced amino acid sequences displaying the highest degree of identity with α-l-fucosidases from Bacteroides thetaiotaomicron, Bacteroides caccae, Phocaeicola vulgatus, Phocaeicola dorei, Ruminococcus gnavus, and Streptococcus parasanguinis. Based on sequence homology, 10 α-l-fucosidase genes were selected for substrate specificity characterization. The α-l-fucosidases Fuc18, Fuc19A, Fuc35B, Fuc39, and Fuc1584 showed hydrolytic activity on α1,3/4-linked fucose present in Lewis blood antigens and the human milk oligosaccharide (HMO) 3-fucosyllactose. In addition, Fuc1584 also hydrolyzed fucosyl-α-1,6-*N*-acetylglucosamine (6FN), a component of the core fucosylation of *N*-glycans. Fuc35A and Fuc193 showed activity on α1,2/3/4/6 linkages from H type-2, Lewis blood antigens, HMOs and 6FN. Fuc30 displayed activity only on α1,6-linked l-fucose, and Fuc5372 showed a preference for α1,2 linkages. Fuc2358 exhibited a broad substrate specificity releasing l-fucose from all the tested free histo-blood group antigens, HMOs, and 6FN. This latest enzyme also displayed activity in glycoconjugates carrying lacto-*N*-fucopentaose II (Le^a^) and lacto-*N*-fucopentaose III (Le^x^) and in the glycoprotein mucin. Fuc18, Fuc19A, and Fuc39 also removed l-fucose from neoglycoproteins and human α-1 acid glycoprotein. These results give insight into the great diversity of α-l-fucosidases from the infant gut microbiota, thus supporting the hypothesis that fucosylated glycans are crucial for shaping the newborn microbiota composition.

**IMPORTANCE** α-l-Fucosyl residues are frequently present in many relevant glycans, such as human milk oligosaccharides (HMOs), histo-blood group antigens (HBGAs), and epitopes on cell surface glycoconjugate receptors. These fucosylated glycans are involved in a number of mammalian physiological processes, including adhesion of pathogens and immune responses. The modulation of l-fucose content in such processes may provide new insights and knowledge regarding molecular interactions and may help to devise new therapeutic strategies. Microbial α-l-fucosidases are exoglycosidases that remove α-l-fucosyl residues from free oligosaccharides and glycoconjugates and can be also used in transglycosylation reactions to synthesize oligosaccharides. In this work, α-l-fucosidases from the GH29 family were identified and characterized from the metagenome of fecal samples of breastfed infants. These enzymes showed different substrate specificities toward HMOs, HBGAs, naturally occurring glycoproteins, and neoglycoproteins. These novel glycosidase enzymes from the breast-fed infant gut microbiota, which resulted in a good source of α-l-fucosidases, have great biotechnological potential.

## INTRODUCTION

L-Fucose (6-deoxy-l-galactose) is a monosaccharide commonly found in soluble glycans such as human milk oligosaccharides (HMOs) and in glycans conjugated to proteins and lipids (1, 2). Fucosyl residues are either α1,2-linked to galactose or α1,3/4/6-linked to *N*-acetylglucosamine (GlcNAc) at the nonreducing end of mammalian glycans (3). All fucosyl linkages with the exception of the α1,6 are present in the histo-blood group antigens (HBGAs), which include the ABO and Lewis antigens. These antigens are displayed as terminal motifs in *N*- and *O*-glycans present in a great variety of tissues, including intestinal mucosa (4). In addition, α1,6-linked l-fucose can be also added to the innermost GlcNAc residue in *N*-glycans constituting the so-called core fucosylation (5). Fucosylated glycoconjugates play fundamental roles in a wide range of physiological processes, including immune responses, receptor signaling, fertilization, tumor metastasis, and host-microbiome interactions (2, 5–8). The l-fucose residue is essential in the molecular interactions occurring in many of these processes. Thus, the fucosyl residues present on the HBGAs mediate contact with many bacterial and viral pathogens (7, 9). As an example, the capsid proteins of important enteric viruses, such as rotaviruses and noroviruses, interact with terminal l-fucose of mucosal HBGAs (10–14). The core l-fucose at the Fc region of IgG antibodies significantly affects its binding to the Fcγ receptor IIIA present on the surface of natural killer cells and macrophages. The removal of the l-fucose residue from the Fc *N*-glycans increases the affinity between IgG and the receptor. As a consequence, the antibody-dependent cellular cytotoxicity activity is enhanced, which is very relevant in cancer immunotherapy (15).

The removal of terminal α-l-fucosyl residues from glycoconjugates and HMOs is catalyzed by α-l-fucosidases, which are classified into two major families, GH29 and GH95, of the Carbohydrate Active Enzymes (CAZy) database (www.cazy.org). GH29 α-l-fucosidases are retaining glycosidases that utilize a double-displacement mechanism, resulting in retention of the anomeric configuration (16, 17), whereas the GH95 family comprise enzymes that follow an inversion mechanism for catalysis (18). GH29 family has been divided into two subfamilies, GH29A and GH29B, according to substrate specificity and phylogenetic relationships (19). The members of the subfamily GH29A (EC 3.2.1.51) have been described to have relatively relaxed substrate specificities, and they can hydrolyze the artificial substrate *p*-nitrophenyl-α-l-fucopyranoside (pNP-Fuc), whereas GH29B (EC 3.2.1.111) members are more specific for α1,3/4 fucosyl linkages and basically do not act on pNP-Fuc (19).

A specific characteristic of human milk is the high level of fucosylated glycans. Depending on host genetics, between 50 and 80% of the HMOs (20) and 75% of the *N*-glycans on milk proteins contain l-fucose (21). These fucosylated HMOs and glycoproteins are crucial for shaping the infant gut microbiota (22). Bacterial species isolated from the gastrointestinal tract are especially adapted to utilize those carbohydrates as fermentable substrate for growth, and they carry in their genomes a complete array of enzymes for their catabolism, including α-l-fucosidases (23, 24). These enzymes have been characterized from several intestinal bacteria, including species belonging to the genera Bacteroides, Bifidobacterium, Clostridium, Lacticaseibacillus, Ruminococcus, and Streptococcus (1, 19, 25–28), but information on the functionality (e.g., substrate specificities) of the many α-l-fucosidases identified by sequence analyses is still scarce. We have previously isolated and characterized three α-l-fucosidases (29), a β-galactosidase (30), and a β-*N*-acetylglucosaminidase (31) from Lacticaseibacillus casei, a species frequently found in infant feces. Recently, our group has also studied two β-galactosidases from an infant gut-associated Bifidobacterium dentium (32).

Since only a small fraction of the total gut microbiota is cultivable, approaches such as metagenomic analyses provide access to greater diversity in protein sequences. In a previous study, we taxonomically analyzed the microbiota composition of fecal samples from breastfed infants (33). The results demonstrated the presence of bacterial genera known to contain putative α-l-fucosidases in their genomes. In the present work, a search of a gut metagenome from those fecal samples has been performed in order to identify and characterize novel α-l-fucosidases. The results revealed a remarkably high number of GH29 α-l-fucosidases present in the infant intestinal environment. In order to get a clearer picture on the capacity of the intestinal microbiota to catabolize fucosylated glycans, the substrate specificity toward naturally occurring free fucosylated oligosaccharides, glycoproteins, and neoglycoproteins was determined for several of these enzymes.

## RESULTS

### Identification of glycosyl hydrolases from infant fecal microbial metagenome.

In order to identify putative glycosidases from the infant intestinal microbiota involved in mucosal and human milk oligosaccharides assimilation, a metagenomic analysis of DNA isolated from fecal samples of breast-fed infants was performed. The RAST-MGRAST server (34, 35) was used for functional annotation of the sequenced metagenome, resulting in the identification of 60,354 sequences that contained predicted proteins with known functions. Using the Cluster of Orthologous Genes (COG) database (36), glycosidases involved in degradation of HMOs such as β-galactosidases, β-*N*-acetylhexosaminidases, α-sialidases, and α-l-fucosidases were identified. Putative β-galactosidases (COG1874, COG2723, and COG3250) were the most abundant glycosidases, with 224 identified enzymes from the GH35, GH42, GH2, and GH1 glycosyl hydrolase families of the CAZy database. They were followed by β-*N*-acetylhexosaminidases (COG3525), which comprised 42 enzymes, all of them members of the GH20 family. Seven sialidases (COG4692 and COG4409) from the GH33 family and 36 putative α-l-fucosidases (COG3669) were also identified. All of the latest enzymes belong to the glycosyl hydrolase family GH29. Members of the other major family of α-l-fucosidases (GH95) were identified using the dbCAN2 server (37), with a total of 27 putative α-l-fucosidases found.

### Sequence analysis and biochemical characterization of GH29 α-l-fucosidases.

We focused on GH29 α-l-fucosidases, where 22 of the 36 open reading frames (ORFs) coding for these putative enzymes showed a complete coding sequence (Table 1). The deduced amino acid sequences of those 22 genes were used in BLAST searches against the nr database (https://blast.ncbi.nlm.nih.gov), and they displayed the best hits (above 98% identity) with α-l-fucosidases annotated in the genomes from the bacterial species Bacteroides thetaiotaomicron, Bacteroides caccae, Ruminococcus gnavus, Phocaeicola vulgatus (formerly Bacteroides vulgatus), Phocaeicola dorei (formerly Bacteroides dorei), and Streptococcus parasanguinis (Table 1). An amino acid percentage of identity matrix (Table S1 in the supplemental material) of the 22 α-l-fucosidases sequences revealed that four groups of enzymes (Fuc1584/Fuc1821, Fuc144/Fuc584/Fuc1327, Fuc11/Fuc19B/Fuc47, and Fuc18/Fuc59/Fuc289/Fuc499) showed sequence identity greater than 69%, which would suggest that the enzymes within a group may have similar substrate specificities. Additionally, pairwise alignments with already-characterized α-l-fucosidases revealed that Fuc11 and Fuc21 showed 99% identity with BT_2970 and BT_2192, respectively, from B. thetaiotaomicron (19). Furthermore, Fuc29 and Fuc144 exhibited 99 and 87% identity with the α-l-fucosidase ATCC_03833 from R. gnavus (27) and BF3242 from Bacteroides fragilis (38), respectively. Considering the homology of these sequences within the predicted α-l-fucosidases listed in Table 1, Fuc18, Fuc19A, Fuc30, Fuc35A, Fuc35B, Fuc39, Fuc193, Fuc1584, Fuc2358, and Fuc5372 were selected for further analysis, since to the best of our knowledge, no proteins homologous to these α-l-fucosidases have yet been characterized.

**TABLE 1:** Putative GH29 α-l-fucosidases found in infant gut microbial metagenome

α-l-Fucosidase	Length (amino acids)	Molecular mass (Da)	Signal peptide	Bacterial species^*a*^	Identity (%)^*b*^
Fuc11	484	55,850	+ (1–29)	Bacteroides thetaiotaomicron (AAO78076)	100
Fuc18	627	71,205	+ (1–21)	Bacteroides thetaiotaomicron (KAB4264829)	100
Fuc19A	471	54,588	+ (1–21)	Bacteroides caccae (MBD9101880)	100
Fuc19B	476	54,780	+ (1–21)	Bacteroides caccae (RHH92811)	100
Fuc21	484	54,563	+ (1–21)	Bacteroides thetaiotaomicron (MCB7009967)	99
Fuc29	435	50,452	−	Ruminococcus gnavus (CCZ67559)	100
Fuc30	464	53,098	+ (1–21)	Bacteroides thetaiotaomicron (KXT30126)	100
Fuc35A	460	53,035	+ (1–20)	Bacteroides caccae (CCZ72424)	100
Fuc35B	452	50,728	+ (1–22)	Bacteroides caccae (CCZ72432)	100
Fuc39	606	68,638	+ (1–19)	Bacteroides thetaiotaomicron (ALJ41003)	100
Fuc47	480	55,826	+ (1–23)	Bacteroides caccae (CCZ72772)	100
Fuc59	625	70,934	+ (1–19)	Bacteroides caccae (RHD46245)	99
Fuc144	438	49,702	+ (1–24)	Bacteroides thetaiotaomicron (AAO76949)	100
Fuc193	461	54,232	+ (1–21)	Bacteroides caccae (KAA5447435)	99
Fuc289	627	70,495	+ (1–19)	Phocaeicola vulgatus (MBV4064515)	100
Fuc499	627	70,655	+ (1–19)	Phocaeicola dorei (MBT1293905)	99
Fuc584	441	50,341	+ (1–25)	Phocaeicola dorei (WP_217775024)	99
Fuc1327	441	50,329	+ (1–25)	Phocaeicola vulgatus (ABR39421)	100
Fuc1584	679	76,692	+ (1–19)	Phocaeicola vulgatus (KAB5455136)	100
Fuc1821	679	76,709	+ (1–19)	Phocaeicola dorei (WP_007839786)	100
Fuc2358	487	56,405	−	Streptococcus parasanguinis (WP_049515056)	98
Fuc5372	449	51,872	+ (1–22)	Phocaeicola dorei (AND21567)	100

a Best hit of BLAST against the nr database of GenBank. The GenBank accession numbers are shown in parentheses.

b Identity to the closest homologue in the nr database of GenBank. The identity percentages correspond to the comparison with the full length of the protein sequence present in the database.

+/−, presence/absence of signal peptide predicted by SignalP 4.1. The numbers in parentheses indicate the amino acid positions that comprise the signal peptide.

**FIG 1:**
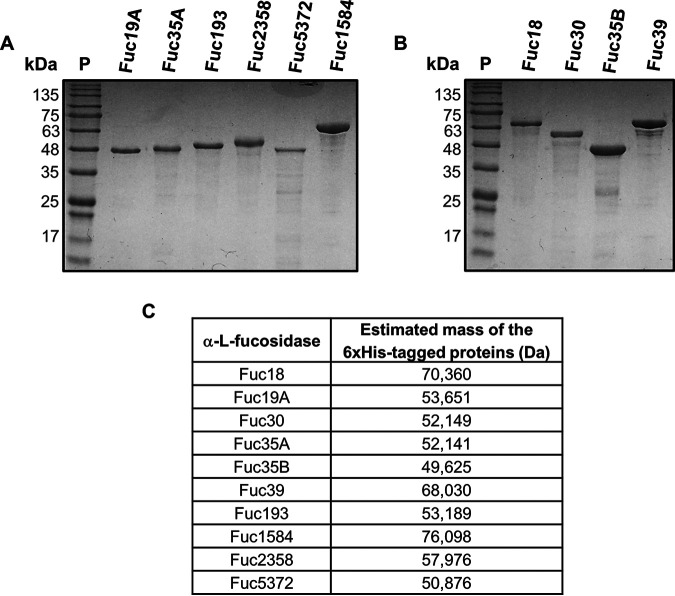
Purified α-l-fucosidases from infant gut metagenome. (A, B) Coomassie brilliant blue-stained 10% SDS-polyacrylamide gel showing the His-tagged proteins α-l-fucosidases GH29 subfamily A (A) and subfamily B (B). (C) Estimated mass of the His_6_-tagged α-l-fucosidases. Lane P, protein standards. The numbers on the left are molecular masses.

**FIG 2:**
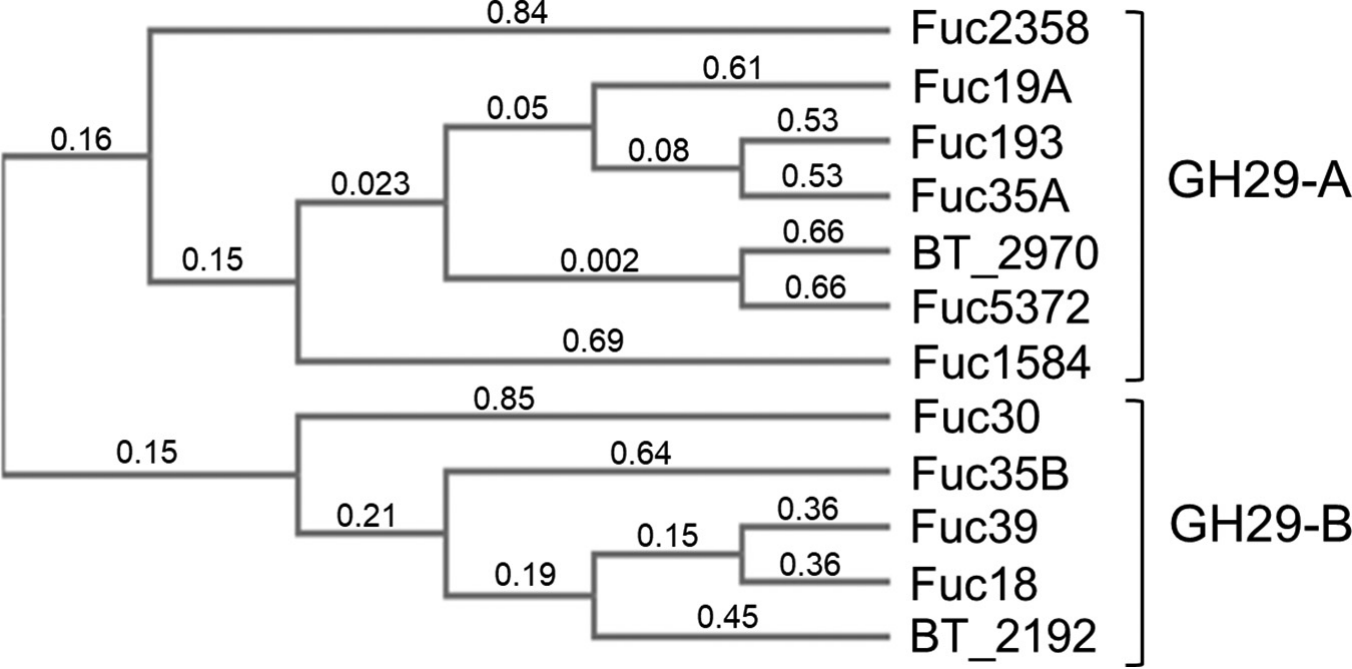
Phylogenetic analysis of α-l-fucosidases studied in this work. The tree shows the metagenome-derived α-l-fucosidases and the previously characterized α-l-fucosidases BT_2970 (GH29 subfamily A) and BT_2192 (GH29 subfamily B) from Bacteroides thetaiotaomicron. Cluster analysis were performed using the unweight pair group method with arithmetic mean (UPGMA). The numbers in the dendrogram provide a measure of sequence distances values between proteins.

SignalP 4.1 (39) predicted the presence of signal peptides in all the selected α-l-fucosidases with the exception of Fuc2358 (Table 1). The 10 putative α-l-fucosidases were expressed in Escherichia coli without signal peptide and purified as His-tagged proteins (calculated molecular weights ranging from 49.62 to 76.09 kDa; Fig. 1). The activity of the purified enzymes was first analyzed against the synthetic substrate *p*-nitrophenyl-α-l-fucopyranoside (*p*NP-fuc). All the enzymes were able to hydrolyze this substrate with the exception of Fuc18 and Fuc39 (Table 2). In agreement with this, both α-l-fucosidases were shown to be phylogenetically close to BT_2192 (Fig. 2), a member of the GH29 subfamily B (19), a subgroup within GH29 previously characterized as unable to cleave *p*NP-fuc. In contrast, Fuc30 and Fuc35B, which are in the same cluster as those three GH29B enzymes, were able to hydrolyze *p*NP-fuc although with low specific activity. Among the tested enzymes, Fuc2358 exhibited the highest specific activity against *p*NP-fuc (Table 2).

**TABLE 2:** Activity and characterization of GH29 α-l-fucosidases from infant gut microbial metagenome

α-l-Fucosidase	sp act (nmol min^−1^ mg protein^−1^)^*a*^	Optimal pH	Optimal temp (°C)
Fuc18	ND		
Fuc19A	2.90 ± 0.21	6.0	60
Fuc30	0.83 ± 0.14	7.0	40
Fuc35A	1.80 ± 0.29	6.5	60
Fuc35B	0.99 ± 0.17	6.5	55
Fuc39	ND		
Fuc193	2.33 ± 0.21	8.0	30
Fuc1584	0.89 ± 0.20	6.0	65
Fuc2358	2,501.39 ± 52.83	6.5	60
Fuc5372	68.47 ± 16.77	6.5	40

a The α-l-fucosidases specific activity was determined with *p*-nitrophenyl-α-l-fucopyranoside as the substrate. Each value represents the mean of three different measurements ± standard deviation. ND, activity not detected.

### Substrate specificity of the α-l-fucosidases from the infant gut microbial metagenome.

**FIG 3:**
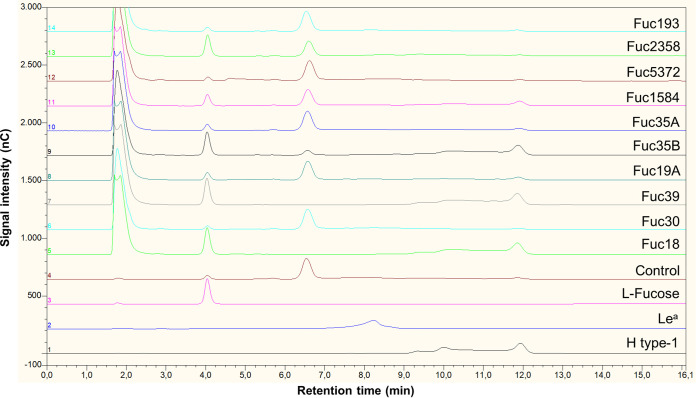
Hydrolysis of Le^b^ by α-l-fucosidases. The figure shows high-performance liquid chromatography (HPLC) chromatograms (Dionex system) of the standard compounds H type-1 (chromatogram 1), Le^a^ (chromatogram 2), l-fucose (chromatogram 3), and control reaction mixture with Le^b^ and without enzyme (chromatogram 4). The reactions with α-l-fucosidases Fuc18, Fuc30, Fuc39, Fuc19A, Fuc35B, Fuc35A, Fuc1584, Fuc5372, Fuc2358, and Fuc193 are shown in chromatograms 5 to 14. H type-1, blood group H type-1 antigen; Le^a^, blood group Lewis a antigen.

**FIG 4:**
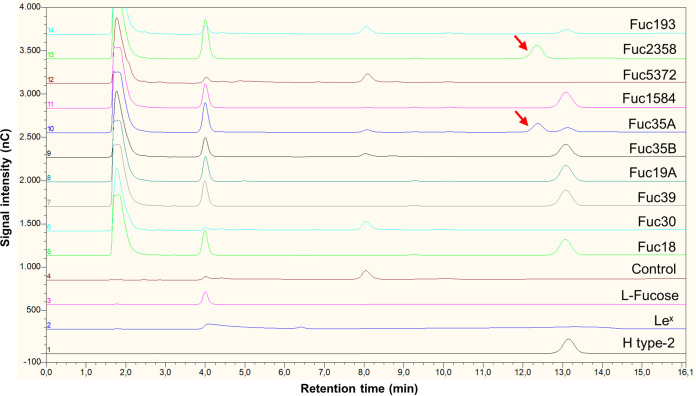
Hydrolysis of blood group Lewis y antigen (Le^y^) by α-l-fucosidases. The figure shows HPLC chromatograms (Dionex system) of the standard compounds H type-2 (chromatogram 1), Le^x^ (chromatogram 2), and l-fucose (chromatogram 3). The control reaction mixture with Le^y^ and without enzyme is represented by chromatogram 4. Reactions with the α-l-fucosidases Fuc18, Fuc30, Fuc39, Fuc19A, Fuc35B, Fuc35A, Fuc1584, Fuc5372, Fuc2358, and Fuc193 are shown in chromatograms 5 to 14. The red arrows indicate the peak of *N*-acetyllactosamine. H type-2, blood group H type-2 antigen; Le^x^, blood group Lewis x antigen.

To test the substrate specificity of the purified α-l-fucosidases, their hydrolase activity was tested on type-1 (containing lacto-*N*-biose; β-d-Gal-(1→3)-β-d-GlcNAc) and type-2 (containing *N*-acetyl lactosamine; β-d-Gal-(1→4)-β-d-GlcNAc) blood antigens, the HMOs 2′-fucosyllactose (2′FL) and 3-fucosyllactose (3FL), and fucosyl-α-1,6-*N*-acetylglucosamine (6FN) that forms part of the core fucosylation of *N*-glycans (Table 3). The α-l-fucosidase Fuc30 displayed activity only on α1,6-linked fucosyl residues, and Fuc5372 showed preference for α1,2 linkages, although low activity for 3FL and 6FN was also observed. Fuc18, Fuc19A, Fuc35B, and Fuc39 showed substrate specificity toward α1,3/4-linked fucosyl residues, and Fuc1584 hydrolyzed α1,3/4/6 linkages. Fuc35A and Fuc193 showed activity on α1,2/3/4/6 linkages, but α1,2-linked residues are hydrolyzed on H type-2 and 2′FL but not on H type-1 glycans. Fuc2358 exhibited a broad substrate specificity releasing fucosyl residues from all the tested glycans. Interestingly, this last enzyme simultaneously hydrolyzed both fucosyl residues from the Lewis b (Le^b^) antigen, since neither H type-1 nor Lewis a (Le^a^) were detected as reaction products (Fig. 3). However, the rest of the α-l-fucosidases acting on this tetrasaccharide removed only the α1,4-linked l-fucose producing H type-1 antigen (Fig. 3). Regarding the activity on Lewis y (Le^y^) antigen, the α-l-fucosidases Fuc2358 and Fuc35A produced *N*-acetyl-d-lactosamine, indicating that these enzymes hydrolyze both α1,2 and α1,3 fucosyl linkages on that tetrasaccharide (Fig. 4). H type-2 antigen was detected as a result of the activity of Fuc18, Fuc19A, Fuc35B, Fuc39, Fuc193, and Fuc1584 on Le^y^, indicating that these enzymes released terminal α1,3 but not α1,2 fucosyl residues from this substrate (Fig. 4).

**TABLE 3:**
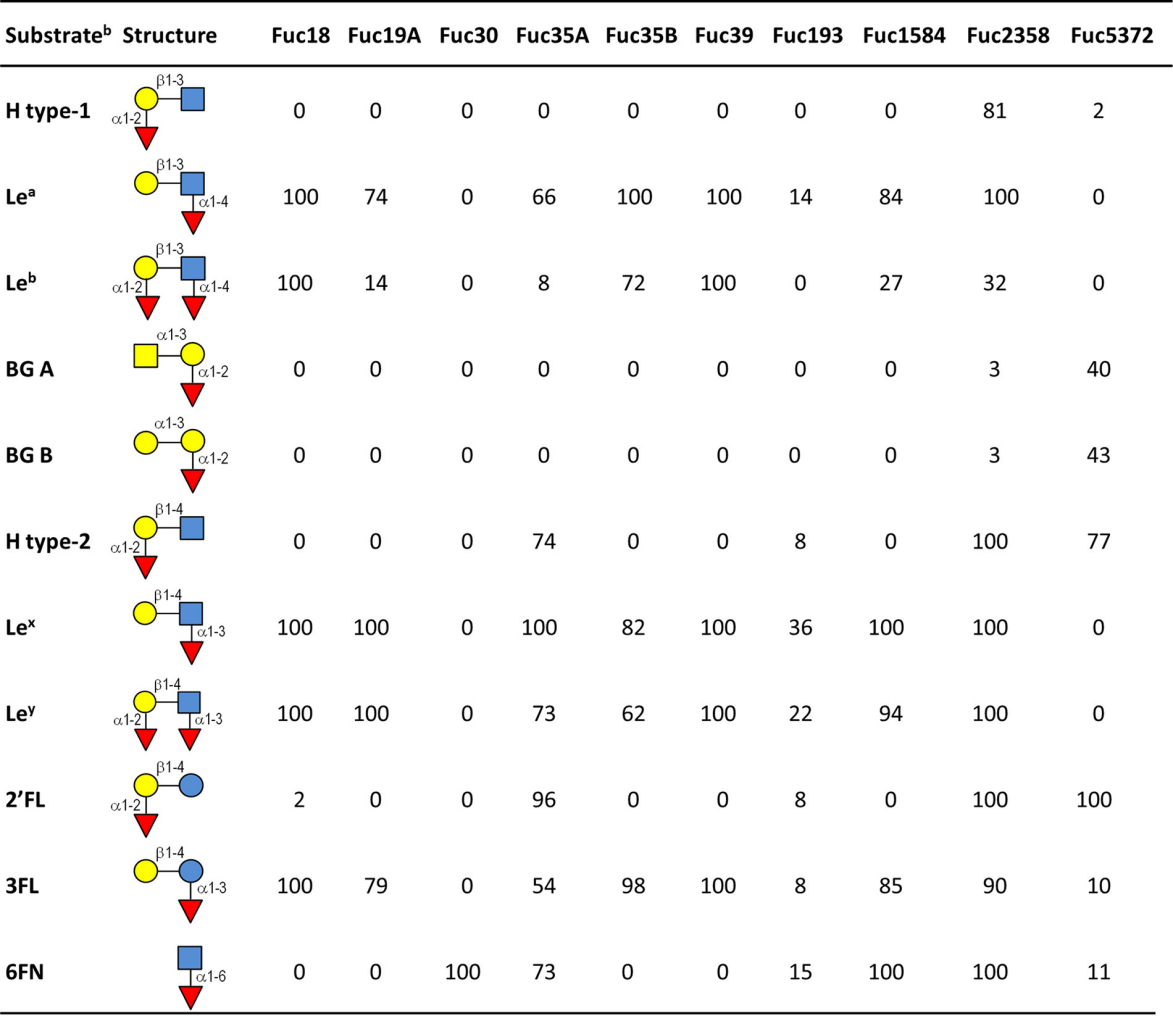
Percentage of hydrolysis of natural fucosyloligosaccharides with the GH29 α-l-fucosidases from infant gut microbial metagenome^*a*^

a The hydrolysis reactions were performed with 2 mM oligosaccharide at 37°C for 16 h.

b H type-1, blood group H type-1 antigen; Le^a^, blood group Lewis a antigen; Le^b^, blood group Lewis b antigen; BG A, blood group A trisaccharide, BG B, blood group B trisaccharide; H type-2, blood group H type-2 antigen; Le^x^, blood group Lewis x antigen; Le^y^, blood group Lewis y antigen; 2′FL, 2′-fucosyllactose; 3FL, 3-fucosyllactose; 6FN, 6-fucosyl-*N*-acetylglucosamine; blue circle, glucose; yellow circle, galactose; blue square, *N*-acetylglucosamine; yellow square, *N*-acetylgalactosamine; red triangle, fucose.

### Activity of the α-l-fucosidases on glycoproteins.

**FIG 5:**
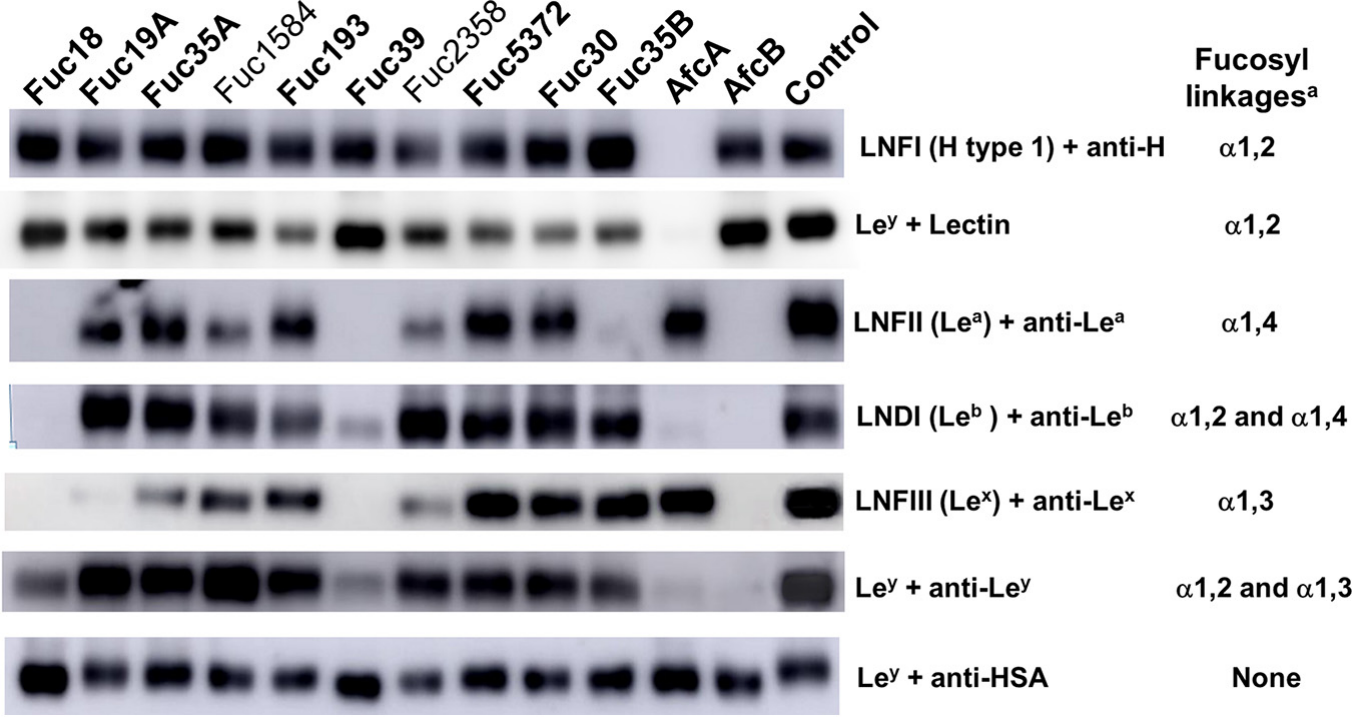
Activity of α-l-fucosidases on fucosylated glycoconjugates. The Western blot shows glycoconjugates (oligosaccharide-human serum albumin [HSA] conjugates) treated with the α-l-fucosidases Fuc18, Fuc19A, Fuc30, Fuc35A, Fuc35B, Fuc39, Fuc193, Fuc1584, Fuc2358, and Fuc5372 studied in this work, as well as the previously characterized AfcA and AfcB. The control included glycoconjugates not treated with enzyme. LNFI, lacto-*N*-fucopentaose I-APD-HSA; LNFII, lacto-*N*-fucopentaose II-APD-HSA; LNDI, lacto-*N*-difucohexaose I-APD-HSA; LNFIII, lacto-*N*-fucopentaose III-APD-HSA; Le^y^, Lewis y-tetrasaccharide-APE-HSA; anti-H, anti-H antibody; anti-Le^a^, anti-Lewis^a^ antibody, anti-Le^b^, anti-Lewis^b^ antibody, anti-Le^x^, anti-Lewis^x^ antibody; anti-Le^y^, anti-Lewis^y^ antibody; Lectin, Ulex europaeus agglutinin (UEA I); APD, acetylphenylenediamine; HSA, human serum albumin. ^a^ Fucosyl linkages recognized by the antibody used in the same row.

The α-l-fucosidases characterized here were further analyzed for activity toward specific glycoconjugates, including lacto-*N*-fucopentaose I (LNFI; α-l-Fuc(1→2)-β-d-Gal-(1→3)-β-d-GlcNAc-(1→3)-β-d-Gal-(1→4)-d-Glc), lacto-*N*-fucopentaose II (LNFP II; Le^a^-lactose; β-d-Gal-(1→3)-[α-l-Fuc-(1→4)]-β-d-GlcNAc-(1→3)-β-d-Gal-(1→4)-d-Glc), lacto-*N*-difucohexaose I (LNDI; Le^b^-lactose; α-l-Fuc-(1→2)-β-d-Gal-(1→3)[α-l-Fuc-(1→4)]-β-d-GlcNAc-(1→3)-β-d-Gal-(1→4)-d-Glc), lacto-*N*-fucopentaose III (LNFIII; Le^x^-lactose; β-d-Gal-(1→4)-[α-l-Fuc-(1→3)]-β-d-GlcNAc-(1→3)-β-d-Gal-(1→4)-d-Glc), and Le^y^-tetrasaccharide (α-Fuc-(1→2)-β-Gal-(1→4)-[α-Fuc-(1→3)]-GlcNAc) bound to human serum albumin (HSA). The products of each enzymatic reaction were analyzed by Western blotting (Fig. 5). The results showed that none of the α-l-fucosidases were able to hydrolyze the α1,2-linked l-fucose from LNFI-HSA (H type-1), including Fuc2358, which acts on soluble H type-1 antigen as described above. The α-l-fucosidase AfcA from Bifidobacterium bifidum (18) was used as a positive control. All the glycoconjugates containing α1,2 fucosyl linkages and treated with this enzyme lost their signals in the Western blots, confirming its specificity on this linkage (18). Fuc2358 showed activity on the glycoconjugates carrying LNFII (Le^a^) and LNFIII (Le^x^) but not on LNDI (Le^b^) and Le^y^. These results indicate that this α-l-fucosidase is able to hydrolyze α1,3- and α1,4-linked l-fucoses from glycoconjugates and suggest that the presence of the α1,2 fucosyl linkages on the Le^b^ and Le^y^ glycoconjugates prevent their hydrolysis. On the contrary, Fuc18 and Fuc39 were able to remove α1,3- and α1,4-linked l-fucoses from all the tested glycoconjugates. The signal patterns of these two enzymes were similar to those obtained with AfcB from B. bifidum, which was used as a positive control for α1,3/4-linkage specificity (40). Fuc19A was also able to hydrolyze the glycoconjugate LNFIII (Le^x^) and Fuc35B hydrolyzed LNFII (Le^a^), indicating that these enzymes have a high specificity for α1,3 and α1,4 fucosyl linkages, respectively, when they are present in glycoconjugates.

The α-l-fucosidases were also assayed for activity toward naturally occurring glycoproteins such as human α-1 acid glycoprotein (AGP), bovine lactoferrin, and porcine mucin by measuring the ability of the enzymes to release l-fucose from these glycosylated proteins (Fig. S1 in the supplemental material). Fuc18, Fuc19A, and Fuc39 were able to release l-fucose from AGP. In agreement with this, the AGP contains sialyl-Le^x^ antigen at the *N*-glycosylation sites (41), and as shown above, these enzymes also showed high hydrolytic activity on the α1,3 fucosyl linkages present on Le^x^ antigen. Fuc2358 acted on porcine mucin removing l-fucose residues. This glycoprotein contains α1,2 fucosyl residues linked to d-galactose (42), and Fuc2358 showed hydrolytic activity on this linkage present in HBGAs and HMOs.

## DISCUSSION

Fucosylated glycans present in human milk contribute to shaping the structure of the breast-fed infant gut microbiota, which can have an important effect on health in the early stages of life but also all through life (43, 44). The ability to utilize those substrates relies on the expression of a wide range of α-l-fucosidases with different linkages specificity by the gut bacteria (1). In addition, the free l-fucose released by bacterial enzymes in the gut lumen may activate l-fucose sensors regulating bacterial intestinal colonization (45). This work reports the analysis of new genes coding GH29 α-l-fucosidases identified through a metagenome analysis of fecal samples of breastfed infants. The detected α-l-fucosidases are probably derived from the bacterial genera Bacteroides, Ruminococcus, Phocaeicola, and Streptococcus. Two α-l-fucosidases, BT_2970 and BT_2192, have been previously characterized in B. thetaiotaomicron (19). Here, three additional novel enzymes from this bacterium (Fuc18, Fuc30, and Fuc39) have been studied, expanding the knowledge on the enzyme repertory for fucosylated compounds catabolism from this human gut symbiont. Fuc18 and Fuc39 have substrate specificities similar to BT_2192; they efficiently hydrolyzed the Lewis antigen trisaccharides and the HMO 3FL. In agreement with these results, it has been recently described that B. thetaiotaomicron is able to ferment low-molecular-weight HMOs, including 3FL (46). Interestingly, Fuc30 showed activity on α1,6-linked fucosyl residues, a characteristic that might utilize B. thetaiotaomicron to scavenge core l-fucose commonly present in mammalian *N*-glycans (47, 48). The variety of α-l-fucosidases may provide B. thetaiotaomicron species with an advantage in colonizing the gut of infants and adults (49, 50). Other species belonging to the Bacteroidaceae family, such as B. caccae, P. vulgatus, and P. dorei, are also consistently present in the gut of newborns (50) and have been described as good consumers of dietary and host-derived glycans (51, 52). However, until now, no α-l-fucosidase enzymes have been characterized from these species. The α-l-fucosidases Fuc19A, Fuc35A, Fuc35B, and Fuc193 present in B. caccae, Fuc1584 in P. vulgatus, and Fuc5372 in P. dorei displayed different substrate specificities. They confirmed the presence of specialized enzymes that would allow these species to utilize fucosylated glycans at the intestinal niche, such as HMOs and epithelial glycans, as sources of energy in this highly competitive environment.

Enzymes belonging to the GH95 family of glycosyl hydrolases, the other major family of α-l-fucosidases, were also identified in the infant fecal microbiome studied here. The deduced amino acid sequences of the 27 genes coding for those glycosidases showed the highest degree of identity with α-l-fucosidases from Bifidobacterium longum subsp. infantis, B. thetaiotaomicron, B. caccae, R. gnavus, P. vulgatus, and P. dorei. Bifidobacterium is one of the main bacterial genera present in the gut of breast-fed infants (53). We have identified here one putative α-l-fucosidase from this genus, which displayed 99% identity with the previously characterized α-l-fucosidase Blon_2335 from B. longum subsp. infantis ATCC 15697 (54). This is in agreement with the presence of the species Bifidobacterium adolescentis, Bifidobacterium longum, and Bifidobacterium pseudolongum in the infant fecal samples analyzed here (33). Most of the sequenced genomes of B. longum subsp. infantis strains contain genes encoding putative α-l-fucosidases (www.cazy.org). However, the sequenced genomes of B. adolescentis strains do not carry genes encoding this type of enzymes, and only one of the six sequenced genomes of B. pseudolongum strains have a putative α-l-fucosidase-encoding gene (www.cazy.org).

An expanded repertory of characterized microbial α-l-fucosidases would not only contribute to understand how gut microbes exploit mucosal carbohydrate resources but could also be a source for important biotechnological applications. The great significance of many fucosylated glycoconjugates in different molecular interactions in mammalians opens the possibility to modulate their levels and specificity of fucosylation for both fundamental and applied research. Thus, a possible approach to determine the function of fucosylation is the enzymatic removal of l-fucose by using specific α-l-fucosidases. The Fuc18, Fuc19A, and Fuc39 enzymes studied here were able to release l-fucose from neoglycoproteins, as well as from AGP, and Fuc2358 removed l-fucose from mucin. Given the substrate specificities toward naturally occurring glycoproteins, those enzymes could constitute tools for research and biotechnological use in defucosylation processes. We showed that Fuc18 and Fuc39 α-l-fucosidases were able to remove α1,3-linked l-fucose from Le^y^ glycoconjugates. As an example, the suppression of the expression of fucosyltransferases FUT1 and FUT4 reduces the synthesis of Le^y^, which is expressed at the cell surface and involved in several physiological and pathological processes, and it results in inhibition of tumor development (55). Also, the α1,6-fucosyl residues present at the human core fucosylation significantly affect the function of glycoproteins such as antibodies (15). IgG without core l-fucose has higher affinity for Fcγ receptor IIIA than core-fucosylated IgG, which is an important feature of therapeutic antibodies (56). The α-l-fucosidases AlfC from L. casei, BfFuc from B. fragilis, and Blon_0248 and Blon_0426 from Bifidobacterium infantis showed high specificity for α1,6-linked l-fucose (29, 57–59). These enzymes are able to act on core fucosylation from antibodies only if the rest of the complex glycan bound to the *N*-linked GlcNAc is removed by endoglycosidases (58–61). No bacterial α-l-fucosidases have been described with the capability to remove the core l-fucose from intact glycosylated IgG. Four additional enzymes, Fuc30, Fuc35A, Fuc1584, and Fuc2358, have been characterized here showing high capacity to hydrolyze α1,6-linked l-fucose from the disaccharide 6FN. These α-l-fucosidases might have applications in the development of therapeutic proteins with modified core fucosylation, although whether or not they are able to act on core fucosylation in glycosylated antibodies needs further analysis.

α-l-Fucosidases from the GH29 family have a retaining double-displacement mechanism that keeps the anomeric configuration (16, 17), and they may catalyze transglycosylation reactions leading to the synthesis of oligosaccharides, such as fucosylated HMOs. The α-l-fucosidases AlfB and AlfC from L. casei were used to synthesize fucosyl-α-1,3-*N*-GlcNAc, 6FN, the glycoaminoacid fucosyl-α1,6-*N*-GlcNAc-Asn, and several 6′-fucosyl-glycans (57, 62). Fucosyl-*N*-GlcNAc disaccharides have also been recently produced using the tranglycosylation activity of α-l-fucosidases isolated from B. fragilis (38). The HMOs 2′FL, 3FL, and lacto-*N*-fucopentaose II have been synthetized in low amounts using the transfucosylation activity of α-l-fucosidases isolated from Thermotoga maritima, Clostridium perfringens, and a soil-derived metagenome library (63, 64). The 10 new GH29 α-l-fucosidases showing different substrate specificities studied in this work might be exploited as biotechnological tools in the synthesis of oligosaccharides.

The α-l-fucosidases characterized here from the gut metagenome of breastfed infants enable the acquisition of basic knowledge about the mechanisms that bacteria utilize to metabolize host-associated glycans and hence adapt to the infant gut environment. The studied enzymes hydrolyze HMOs, terminal blood group antigens that are commonly present in the glycans of glycoproteins and glycolipids, and α1,6-fucosyl residues found in the core of *N*-glycans. These enzymes could have great potential for use in various biotechnological applications. Future work, including metatranscriptomic analysis of the infant gut microbiota, should provide additional understanding of the role of α-l-fucosidase enzymes in the degradation of human milk oligosaccharides and intestinal glycoconjugates.

## MATERIALS AND METHODS

### Fecal samples and microbial DNA extraction.

Stool samples from four infants exclusively breast-fed and between the ages of 1 and 3 months were collected. They were immediately stored at 4°C and then kept at –80°C within the next 12 h. A pooled fecal sample (one sample from each infant) was used to isolate total DNA using the MasterPure Complete DNA and RNA purification kit (Epicentre). The manufacturer’s instructions were followed with some modifications, including incubation with lysozyme (40 μg) and mutanolysin (10 U) at 37°C for 60 min and a cell disruption step using 0.1-mm-diameter glass beads (FastPrep 24-5 G Homogenizer, MP Biomedicals, CA, USA). DNA concentration was measured using a Qubit 2.0 fluorometer (Life Technology).

The use of human samples was approved by the Ethical Committee for Human Research of the University of Valencia, with registration number H1544010468380. Written informed consent was obtained from a parent of each of the subjects.

### Metagenomic analysis of the pooled fecal sample.

A genomic DNA library was constructed using a TruSeq DNA library kit. A Bioanalyzer DNA 1000 chip (Agilent Technologies) was used to verify the amplicons’ size. The library was sequenced using a 2 × 300 bp paired-end run (MiSeq reagent kit v3) on a MiSeq Sequencer according to the manufacturer’s instructions (Illumina) by the Central Service of Research Support of the University of Valencia (Spain). Raw reads generated from the MiSeq Sequencer were checked for quality, adapter-trimmed, and filtered using the AfterQC (65) and FastQC version 0.11.8 (http://www.bioinformatics.babraham.ac.uk) tools. Metagenomic assembling was carried out using metaSPAdes (66). The RAST-MGRAST web server was used for functional profiling of the sequenced metagenome (34, 35). ORFs were annotated using the Cluster of Orthologous Genes (COG) database (36). The dbCAN2 meta server (37) was used to predict the genes encoding glycosyl hydrolases of the family GH95 defined by the CAZy database.

The percentage of amino acid identity matrix of the putative α-l-fucosidases was calculated with the CLUSTAL Omega sequence alignment program from the EMBL-EBI (67). The sequences of the α-l-fucosidases were subjected to the multiple alignments by the ClustalW and ClustalX version 2 (68) and the analysis tool web services from the EMBL-EBI (67). Cluster analyses were performed using the unweight pair group method with arithmetic mean (UPGMA).

### Expression and purification of His-tagged α-l-fucosidases.

Ten putative α-l-fucosidase genes were amplified by PCR with the Phusion high-fidelity DNA polymerase (Thermo Fisher Scientific) using total DNA isolated from the pooled fecal sample and specific primers (Table S2 in the supplemental material). The PCR fragments were digested with specific restriction enzymes and cloned into pQE80 vector (Qiagen) cleaved with the same enzymes. The resulting plasmids pQEfuc18, pQEfuc19A, pQEfuc30, pQEfuc35A, pQEfuc35B, pQEfuc39, pQEfuc193, pQEfuc1584, pQEfuc2358, and pQEfuc5372 (Table 4) were used to transform E. coli DH10B by electroporation with a Gene Pulser apparatus (Bio-Rad) as recommended by the manufacturer. E. coli transformants were selected on Luria-Bertani (LB) agar plates at 37°C with ampicillin (100 μg mL^−1^). DNA sequencing was performed by Eurofins Genomics (http://www.eurofinsgenomics.com) to confirm the correct sequence of the inserts. One clone of each, PE179 (pQEfuc18), PE180 (pQEfuc19A), PE181 (pQEfuc30), PE182 (pQEfuc35A), PE183 (pQEfuc35B), PE184 (pQEfuc39), PE185 (pQEfuc193), PE186 (pQEfuc1584), PE187 (pQEfuc2358), and PE188 (pQEfuc5372) (Table 4), was cultured overnight in 250 mL of LB at 25°C under agitation. When the cultures reached an optical density at 595 nm (OD_595_) of 0.7, isopropyl-β-d-thiogalactopyranoside was added at 0.2 mM for protein induction, and incubation was continued for 5 h. The cell extracts were prepared as previously described (30), and they were loaded onto Ni-Sepharose 6 Fast Flow columns (HisTrap FF crude columns, GE Healthcare). The recombinant proteins were purified using an Äkta Prime FPLC system (GE Healthcare) as previously described (30) and kept at –80°C in 100 mM Tris-HCl buffer, pH 7.5, containing 20% glycerol. Protein assay dye reagent concentrate (Bio-Rad) was used to determine the protein concentration.

**TABLE 4:** Strains and plasmids used in this study

Strain or plasmid	Relevant genotype or properties	Source
E. coli strains		
DH10B	F-*endA1 recA1 galE15 galK16 nupG rpsL* Δl*acX74* Φ80l*acZ*ΔM15 *araD139* Δ(*ara, leu*)7697 *mcrA* Δ(*mrr-hsdRMS-mcrBC*) λ	Invitrogen
PE179	DH10B containing pQEfuc18	This work
PE180	DH10B containing pQEfuc19A	This work
PE181	DH10B containing pQEfuc30	This work
PE182	DH10B containing pQEfuc35A	This work
PE183	DH10B containing pQEfuc35B	This work
PE184	DH10B containing pQEfuc39	This work
PE185	DH10B containing pQEfuc193	This work
PE186	DH10B containing pQEfuc1584	This work
PE187	DH10B containing pQEfuc2358	This work
PE188	DH10B containing pQEfuc5372	This work
Plasmids		
pQE80	E. coli expression vector; Amp^R^	Qiagen
pQEfuc18	pQE80 containing *fuc18*-coding region	This work
pQEfuc19A	pQE80 containing *fuc19A*-coding region	This work
pQEfuc30	pQE80 containing *fuc30*-coding region	This work
pQEfuc35A	pQE80 containing *fuc35A*-coding region	This work
pQEfuc35B	pQE80 containing *fuc35B*-coding region	This work
pQEfuc39	pQE80 containing *fuc39*-coding region	This work
pQEfuc193	pQE80 containing *fuc193*-coding region	This work
pQEfuc1584	pQE80 containing *fuc1584*-coding region	This work
pQEfuc2358	pQE80 containing *fuc2358*-coding region	This work
pQEfuc5372	pQE80 containing *fuc5372*-coding region	This work

### Biochemical characterization of α-l-fucosidases.

Reaction mixtures (100 μL) in 96-well plates containing *p*-NP-α-l-fucopyranoside (*p*NP-fuc) 5 mM in 100 mM Tris-HCl buffer, pH 7.0, were initiated by adding 10 μg of enzyme. The activity was assayed at 37°C, and the *p*-nitrophenol released was measured by following continuous changes in absorbance at 404 nm using a POLARstar Omega microplate reader (BMG Labtech, Offenburg, Germany). The optimal pH was determined using 100 mM phosphate-citrate buffer (pH 3.0 to 8.5), and the optimal temperature reaction was analyzed in a range from 25 to 70°C at the optimal pH.

### Evaluation of hydrolytic activity on natural fucosyloligosaccharides.

The ability of the α-l-fucosidases to hydrolyze natural fucosyloligosaccharides (Biosynth Carbosynth) was assayed using several substrates listed in Table 3. The reaction mixtures (10 μL) were performed at 37°C during 16 h using 2 mM substrate in phosphate-buffered saline (PBS) (Sigma) and 2 μg of enzyme. After incubation, the reaction mixtures were diluted 10 times with deionized water and analyzed by high-performance liquid chromatography using an ICS3000 chromatographic system (Dionex) and a CarboPac PA100 column with pulsed amperometric detection. A gradient of 10 to 100 mM NaOH for 30 min was used at 27°C and at a flow rate of 1 mL/min. Reaction products were confirmed by comparing their retention times with those of standard carbohydrates.

### Evaluation of hydrolytic activity on neoglycoproteins.

The ability of the α-l-fucosidases to hydrolyze glycoconjugates was assayed using several neoglycoproteins (IsoSep) listed in Fig. 5. The reaction mixtures (25 μL) containing 5 μg of α-l-fucosidase and 1 μg of substrate in PBS were incubated at 37°C for 20 h. After incubation, 5-μL samples of each reaction were run in 12% SDS-PAGE and transferred to nitrocellulose membranes for Western blot analysis. The membranes were washed with PBS and blocked with PBS containing 0.05% Tween 20 (PBS-T) and 5% skim milk for 1 h at room temperature with gentle agitation. After blocking, the membranes were washed two times with PBS-T and incubated with the appropriate antibody (anti-H antibody [catalog no. 922102, BioLegend]; anti-Lewis^a^ antibody [catalog no. 922202, BioLegend]; anti-Lewis^b^ antibody [catalog no. SAB4700761, Sigma]; anti-Lewis^x^ antibody [catalog no. 912901, BioLegend]; and anti-Lewis^y^ antibody [catalog no. 912501, BioLegend]; anti-HAS antibody conjugated to horseradish peroxidase [HRP] [catalog no. ab8941, Abcam] or lectin [Ulex europaeus agglutinin conjugated to HRP; catalog no. L8146; Sigma]) (Fig. 5) in PBS-T overnight at 4°C. The membranes were washed three times with PBS-T and incubated with HRP-anti mouse antibody diluted 1:10,000 (Millipore) in PBS-T for 1 h at room temperature when needed. Finally, the membranes were washed three times with PBS-T, and the immunoblots were developed with the Immobilon Western reagent (Millipore).

### Evaluation of hydrolytic activity on natural glycoproteins.

The α-l-fucosidases were assayed for activity toward human α-1 acid glycoprotein (AGP) (Sigma-Aldrich), bovine lactoferrin (Friesland Campina Domo), and porcine mucin (Sigma-Aldrich). The reaction mixtures (10 μL) were performed at 37°C for 16 h using 0.5 μg μL^−1^ substrate in PBS (Sigma) and 2 μg of enzyme. After incubation, the reaction mixtures were diluted 10 times with deionized water and analyzed by chromatography using the Dionex system and column described above.

### Data availability.

Raw Sequence data files are available in the NCBI (http://www.ncbi.nlm.nih.gov/sra) Sequence Read Archive (SRA) under Bioproject number PRJNA814838. The sequences of the genes encoding the α-l-fucosidases characterized here have been deposited at the GenBank database under the accession numbers ON170361 to ON170370.
