# Supplementary material for: Infant Gut Microbial Metagenome Mining of α-l-Fucosidases with Activity on Fucosylated Human Milk Oligosaccharides and Glycoconjugates
Source: Microbiol Spectr. 2022 Aug 9;10(4):e01775-22. doi: 10.1128/spectrum.01775-22 (PMC9430343; doi:10.1128/spectrum.01775-22)
Supplement: Supplemental file 1 — Supplemental material. Download spectrum.01775-22-s0001.pdf, PDF file, 0.1 MB [file spectrum.01775-22-s0001.pdf]

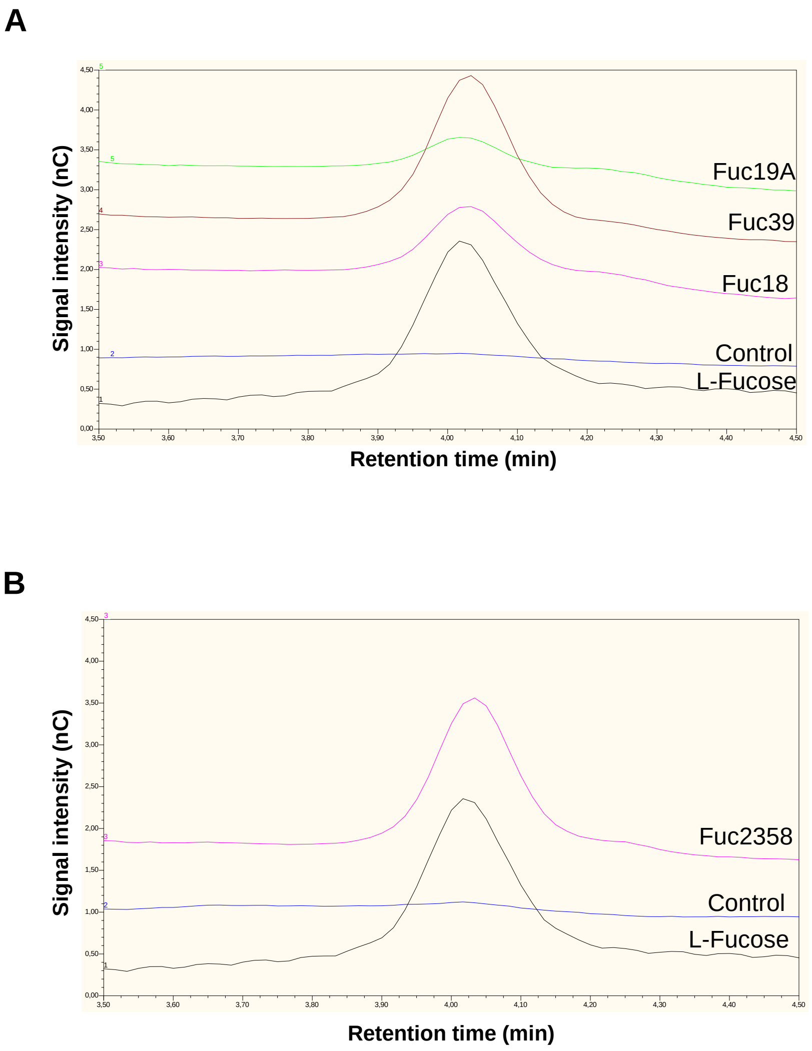

Figure S1. Hydrolysis of human α-1 acid glycoprotein (A) and porcine mucin (B) by α-L-fucosidases. HPLC chromatograms (Dionex system) of the standard compound L-fucose (chromatograms 1A and 1B). Control, reaction mixture with human α-1 acid glycoprotein (chromatogram 2A) or porcine mucin (chromatogram 2B) and without enzyme. Reactions mixtures with human α-1 acid glycoprotein as substrate and α-L-fucosidases Fuc18, Fuc39 and Fuc19A (chromatograms 3A-5A). Reactions mixture with porcine mucin as substrate and α-L-fucosidase Fuc2358 (chromatogram 3B).

TABLE S1. Percent amino acid identity matrix of the α-L-fucosidases GH29 found in infant gut microbial metagenome[a]

	Fuc2358	Fuc1821	Fuc1584	Fuc5372	Fuc35A	Fuc144	Fuc584	Fuc1327	Fuc19A	Fuc47	Fuc11	Fuc19B	Fuc29	Fuc193	Fuc30	Fuc35B	Fuc39	Fuc21	Fuc18	Fuc59	Fuc289	Fuc499
1: Fuc2358	100.00	24.63	24.38	24.35	27.45	27.72	28.42	28.42	23.38	20.90	21.49	21.22	24.67	21.36	17.46	18.39	20.40	18.54	17.56	17.85	18.93	18.93
2: Fuc1821	24.63	100.00	97.94	26.99	31.53	31.99	31.31	31.31	25.92	27.42	26.97	26.97	29.56	28.68	23.71	21.31	21.97	20.20	23.20	23.85	24.05	23.45
3: Fuc1584	24.38	97.94	100.00	27.71	31.29	32.24	31.57	31.57	25.92	27.42	26.97	26.97	29.31	28.18	23.45	21.04	21.97	19.95	22.60	23.85	24.25	23.65
4: Fuc5372	24.35	26.99	27.71	100.00	32.27	34.51	33.17	32.92	26.94	32.56	31.69	32.47	31.47	30.18	19.68	22.50	21.04	17.38	17.79	19.24	18.65	18.65
5: Fuc35A	27.45	31.53	31.29	32.27	100.00	38.15	36.16	36.16	28.32	30.36	29.82	28.53	31.15	31.74	26.69	20.34	24.21	18.26	24.43	25.28	23.23	22.66
6: Fuc144	27.72	31.99	32.24	34.51	38.15	100.00	69.68	69.91	28.46	27.25	29.09	28.05	32.18	29.15	26.94	23.50	26.54	20.61	24.65	26.40	24.93	24.93
7: Fuc584	28.42	31.31	31.57	33.17	36.16	69.68	100.00	97.05	27.48	26.46	27.58	26.55	32.01	28.54	24.52	21.91	24.65	20.16	22.31	22.99	22.10	21.27
8: Fuc1327	28.42	31.31	31.57	32.92	36.16	69.91	97.05	100.00	27.23	26.72	27.84	26.55	31.75	28.54	24.52	22.75	24.65	20.16	23.14	23.27	22.93	22.10
9: Fuc19A	23.38	25.92	25.92	26.94	28.32	28.46	27.48	27.23	100.00	25.52	26.74	26.28	32.33	32.32	24.23	21.23	22.01	22.59	22.50	22.07	22.84	22.28
10: Fuc47	20.90	27.42	27.42	32.56	30.36	27.25	26.46	26.72	25.52	100.00	76.37	76.37	32.32	30.90	23.16	18.50	21.67	19.31	22.67	24.66	21.93	22.19
11: Fuc11	21.49	26.97	26.97	31.69	29.82	29.09	27.58	27.84	26.74	76.37	100.00	92.65	33.00	30.79	24.38	18.98	24.93	19.84	22.97	25.27	24.12	24.39
12: Fuc19B	21.22	26.97	26.97	32.47	28.53	28.05	26.55	26.55	26.28	76.37	92.65	100.00	32.49	30.07	23.63	18.33	22.89	19.84	21.89	23.64	22.76	23.04
13: Fuc29	24.67	29.56	29.31	31.47	31.15	32.18	32.01	31.75	32.33	32.32	33.00	32.49	100.00	36.74	24.93	18.98	19.94	23.06	20.90	20.90	20.90	20.62
14: Fuc193	21.36	28.68	28.18	30.18	31.74	29.15	28.54	28.54	32.32	30.90	30.79	30.07	36.74	100.00	24.32	21.53	24.38	23.26	24.33	22.85	25.20	24.93
15: Fuc30	17.46	23.71	23.45	19.68	26.69	26.94	24.52	24.52	24.23	23.16	24.38	23.63	24.93	24.32	100.00	24.81	25.00	24.77	23.80	23.62	24.77	24.54
16: Fuc35B	18.39	21.31	21.04	22.50	20.34	23.50	21.91	22.75	21.23	18.50	18.98	18.33	18.98	21.53	24.81	100.00	31.47	33.33	32.08	32.94	31.46	31.22
17: Fuc39	20.40	21.97	21.97	21.04	24.21	26.54	24.65	24.65	22.01	21.67	24.93	22.89	19.94	24.38	25.00	31.47	100.00	37.75	41.06	42.65	40.99	40.99
18: Fuc21	18.54	20.20	19.95	17.38	18.26	20.61	20.16	20.16	22.59	19.31	19.84	19.84	23.06	23.26	24.77	33.33	37.75	100.00	40.30	40.38	38.40	37.97
19: Fuc18	17.56	23.20	22.60	17.79	24.43	24.65	22.31	23.14	22.50	22.67	22.97	21.89	20.90	24.33	23.80	32.08	41.06	40.30	100.00	77.40	72.80	72.96
20: Fuc59	17.85	23.85	23.85	19.24	25.28	26.40	22.99	23.27	22.07	24.66	25.27	23.64	20.90	22.85	23.62	32.94	42.65	40.38	77.40	100.00	80.35	80.51
21: Fuc289	18.93	24.05	24.25	18.65	23.23	24.93	22.10	22.93	22.84	21.93	24.12	22.76	20.90	25.20	24.77	31.46	40.99	38.40	72.80	80.35	100.00	96.49
22: Fuc499	18.93	23.45	23.65	18.65	22.66	24.93	21.27	22.10	22.28	22.19	24.39	23.04	20.62	24.93	24.54	31.22	40.99	37.97	72.96	80.51	96.49	100.00

[a]α-L-fucosidases with amino acid identity above 69 % are labeled with the same color.

TABLE S2. Primers used in this study

Name	Sequence
Fuc18BamHI	5'-TTTTGGATCCTGTCAGAGTGTTTCGGCAC
Fuc18HindIII	5'-TTTTAAGCTTCTATCGACACCCAATTTCCG
Fuc19ABamHI	5'-TTTTGGATCCCAACACACATTTGTACATAAAC
Fuc19APstI	5'-TTTTCTGCAGTCACTTCTTGTTTTGTTTCGG
Fuc30BamHI	5'-TTTTGGATCCCAAACTAAGTCGTTGCACC
Fuc30HindIII	5'-TTTTAAGCTTTCATCTTCTCTCACAATATAC
Fuc35ABamHI	5'-TTTTGGATCCACTACCGAAGACAATTTGCAT
Fuc35APstI	5'-TTTTCTGCAGTCAGAAATATTCTACTTTTAGC
Fuc35BBamHI	5'-TTTTGGATCCCAAGAGAAAATAATTGTATGTC
Fuc35BHindIII	5'-TTTTAAGCTTTTAATATATTGCGAAAGACTTTA
Fuc39BamHI	5'-TTTTGGATCCTGTGGACTGACGAAAGAAAAC
Fuc39HindIII	5'-TTTTAAGCTTTCAAGGAGCTACGGTCAC
Fuc193BamHI	5'-TTTTGGATCCACACATATTGTTCAAAATCAATA
Fuc193HindIII	5'-TTTTAAGCTTCTACTCCAATATAAGTTCTA
Fuc1584BamHI	5'-TTTTGGATCCCAAATCTCGGAAAAAGCATTTG
Fuc1584PstI	5'-TTTTCTGCAGTTATTTTGCCTCTACCTTCAG
Fuc2358SphI	5'-TTTTGCATGCATGATAAGCCTTGAAGAGATTG
Fuc2358HindIII	5'-TTTTAAGCTTCTAAAAGCTGATGCGCAAAAC
Fuc5372BamHI	5'-CTGGGATCCCAAGATACCCTGCAAAATGAGACC
Fuc5372HindIII	5'-ATTAAGCTTGGCTGCAGGTCGACCCTAGCTAGG
